# Metoclopramide induced acute dystonic reaction: a case report

**DOI:** 10.1186/s13104-016-2342-6

**Published:** 2017-01-07

**Authors:** Frank-Leonel Tianyi, Valirie Ndip Agbor, Tsi Njim

**Affiliations:** 1 Sub-Divisional hospital Mayo Darley, Mayo Darley, Cameroon; 2 Ibal sub-Divisional hospital, Oku, North west region Cameroon; 3 Centre for Tropical Medicine and Global Health, Nuffield Department of Medicine, University of Oxford, Oxfordshire, UK; 4 Health and Human Development Research Group (2HD), Douala, Cameroon

**Keywords:** Metoclopramide, Dystonic reactions, Chlorpheniramine

## Abstract

**Background:**

Metoclopramide is a commonly used anti-emetic drug known to cause extrapyramidal symptoms as adverse effects, amongst which are dystonic reactions. These reactions are more frequent at high doses of metoclopramide, in female patients, children and adults less than 30 years of age. We hereby present the case of a 16 year old female who had dystonic reactions from metoclopramide, highlighting its unpredictable nature and the shortcomings of the management in resource-limited settings.

**Case presentation:**

A 16 year old female Muslim from the Extreme North of Cameroon with no significant past history, was treated for severe malaria and associated refractory vomiting using intravenous quinine and metoclopramide respectively. She developed dystonic reactions after being administered her second dose of metoclopramide. The drug was discontinued and she was administered 8 mg of chlorpheniramine by mouth. Her symptoms resolved after 4 h. She was discharged 2 days later with no further complaints.

**Conclusions:**

Metoclopramide causes dystonic reactions which are often unpredictable and is frequently prescribed by health providers. This creates an environment of anxiety for the patient and the caregiver, and can result in life threatening consequences. Patients on metoclopramide should be monitored closely to detect these reactions early, and health facilities should be equipped to cope with the adverse effects before administration.

## Background

Dystonia is a movement disorder characterized by involuntary, sustained or spasmodic contractions of muscle groups, resulting in twisting, repetitive and abnormal positions [1]. Acute dystonic reactions are the most common type of extrapyramidal reactions associated with the use of metoclopramide [2]. Standard management involves discontinuation of the drug and rapid intravenous or intramuscular administration of an anticholinergic or antihistaminic drug [3]. We present a case of an acute dystonic reaction in a resource limited setting which was managed with an oral antihistaminic drug.

## Case presentation

A 16 year old female Muslim from the Extreme North of Cameroon with no significant past history was treated for severe malaria using intravenous quinine. Due to refractory nausea and vomiting she was also put on intravenous metoclopramide, 10 mg three times a day. A few minutes after administration of the second dose of metoclopramide, she complained of pain and stiffness of the neck and an inability to keep her tongue in her mouth. On examination, she was conscious and anxious with a blood pressure of 124/78 mmHg, pulse rate of 90 beats/minute, respiratory rate of 20 breaths/minute and temperature of 37.3 °C. Her neck was slightly arched backwards and her tongue was hanging out. There was no accumulation of saliva in her mouth and she could swallow, but with difficulty. There were no signs of respiratory distress. The rest of her examination was unremarkable. A diagnosis of metoclopramide induced acute dystonic reaction was made. She was administered 8 mg of oral chlorpheniramine. Her dystonic symptoms subsided 4 h later, as did the nausea and vomiting. She continued her antimalarial treatment and the metoclopramide was discontinued. She did not have any further dystonic symptoms. She was discharged 2 days later and was advised to avoid taking metoclopramide. Follow-up visit 2 weeks later was unremarkable.

## Conclusions

Metoclopramide hydrochloride is a dopamine receptor antagonist used in the management of gastrointestinal disorders such as nausea, vomiting and gastroparesis [4]. The antagonistic activity of metoclopramide at the central and peripheral dopamine receptors gives rise to its anti-emetic properties. [5, 6]. Dopamine causes nausea and vomiting by stimulating the medullary chemoreceptor trigger zone. The antagonistic effect of metoclopramide on dopamine receptors in the basal ganglia [5, 7], causes an alteration in the dopaminergic-cholinergic balance, resulting in a deficit in central dopamine transmission; and hence an excess release of acetylcholine over dopamine [5–7]. The incidence of metoclopramide-induced dystonic reactions in studies carried out in the developed world is reported to be 1:500 patients [2, 4]. In the United States, over 7 million prescriptions were made for metoclopramide in 2004, which is over a threefold increase compared to the number of prescriptions 10 years before [8]. In the developing world, very little research has been performed on metoclopramide-induced reactions, which is surprising given the observed increase in prescriptions and availability as an over the counter anti-emetic drug. The incidence of these reactions is underestimated due to lack of data collection and under-reporting of cases. Female patients, children, adults less than 30 years of age, and patients receiving high doses of metoclopramide have higher chances of developing dystonic reactions [1, 5, 7, 9]. Our patient was a 16-year-old female, so compared to the general population, she had a slightly increased risk of having a dystonic reaction to metoclopramide.

Following administration of metoclopramide, symptoms can take up to 36 h to appear, and are usually in the form of involuntary limb movements, facial grimacing, torticollis, oculogyric crisis, rhythmic protrusion of the tongue, bulbar type of speech, trismus, opisthotonus and rarely stridor and dyspnea which result from laryngospasm [5, 10].

Standard management involves discontinuation of metoclopramide and administration of injectable anticholinergic or antihistaminic drugs, most often, benztropine and diphenhydramine. The intravenous route is the route of choice with signs and symptoms resolving within 10 min. The intramuscular route is an alternative in case of inability to obtain an intravenous line, but it takes 30 min to be absorbed [3]. The oral route is the most unreliable, as oral chlorpheniramine undergoes extensive gut first pass metabolism with a bioavailability of as low as 25% and a mean peak time of up to 4 h [11].

In this case, our patient had torticollis and rhythmic tongue protrusion. She could not benefit from the recommended injectable anticholinergic or antihistaminic treatment due to unavailability of these drugs. The only formulation of an antihistaminic drug present at our institution at the time was oral chlorpheniramine. Given the urgency of the situation, the healthcare team could not waste time to arrange for referral with bad roads or look for appropriate drugs which are often sparsely distributed in pharmacies. Hence, she was administered oral chlorpheniramine which has a low bioavailability and a slow onset of action compared to the injectable forms, and it is not too reliable in conditions which interfere with swallowing [5, 11]. Also, some cases of severe tongue protrusion dystonia are unresponsive to oral medications [12]. This highlights the challenges in resource limited settings, as not only are essential drugs lacking, but they lack basic resuscitation facilities, hence they are not equipped to handle the adverse effect of drugs administered in the health centers.

This case report demonstrates how unpredictable dystonic reactions can be, the importance of considering dystonic reactions as a possible side-effect each time metoclopramide is administered, and the need to be prepared to treat acute dystonia in facilities in which it is administered. It also highlights the shortcomings of hospitals in resource-limited settings for managing this adverse reaction. This is a critical issue because the manifestations of an acute dystonic reaction following the administration of an anti-emetic may be life-threatening in rare instances [1, 13, 14]. Hence, health policies should dictate that institutions which administer metoclopramide and other dystonia-inducing drugs should be well equipped to handle their extrapyramidal adverse effects.

Metoclopramide is a useful drug in the management of nausea and vomiting related disorders and is prescribed by health personnel at different levels. They cause dystonic reactions which do not only create an environment of anxiety for the patient and the care-giver, but could also be life threatening. Patients on metoclopramide should be monitored closely to detect these reactions early, and health facilities should be equipped to cope with the adverse effects before serving the drugs.
